# Prevalence of *Staphylococcus aureus* protein A (*spa*) mutants in the community and hospitals in Oxfordshire

**DOI:** 10.1186/1471-2180-14-63

**Published:** 2014-03-12

**Authors:** Antonina A Votintseva, Rowena Fung, Ruth R Miller, Kyle Knox, Heather Godwin, David H Wyllie, Rory Bowden, Derrick W Crook, A Sarah Walker

**Affiliations:** 1 Nuffield Department of Clinical Medicine, University of Oxford, John Radcliffe Hospital, Level 7, Room 7724, Oxford OX3 9DU, United Kingdom; 2 National Institute for Health Research (NIHR) Oxford Biomedical Research Centre, John Radcliffe Hospital, Oxford OX3 9DU, United Kingdom; 3 Department of Molecular Microbiology, John Innes Centre, Norwich Research Park, Norwich NR4 7UH, United Kingdom; 4 School of Population and Public Health, University of British Columbia, British Columbia Centre for Disease Control, 655 West 12th Avenue, Vancouver, BC V5Z 4R4, Canada; 5 The Jenner Institute, University of Oxford, Roosevelt Drive, Oxford OX3 7DQ, United Kingdom; 6 Wellcome Trust Centre for Human Genetics, University of Oxford, Roosevelt Drive, Oxford OX3 7BN, United Kingdom; 7 Gasteroenterology Research Office, Great Ormond Street Hospital for Children NHS Foundation Trust, Great Ormond Street, London WC1N 3JH, United Kingdom; 8 Nuffield Department of Primary Care Health Sciences, University of Oxford, Oxford OX1 2ET, United Kingdom

**Keywords:** *Staphylococcus aureus*, Spa-typing, Spa-gene, Deletions, Non-typeable isolates, Community and hospital strains

## Abstract

**Background:**

Staphylococcal protein A (*spa*) is an important virulence factor which enables *Staphylococcus aureus* to evade host immune responses. Genotypes known as “*spa*-types”, based on highly variable Xr region sequences of the *spa*-gene, are frequently used to classify strains. A weakness of current *spa*-typing primers is that rearrangements in the IgG-binding region of the gene cause 1-2% of strains to be designated as “non-typeable”.

**Results:**

We developed an improved primer which enabled sequencing of all strains, containing any type of genetic rearrangement, in a large study among community carriers and hospital inpatients in Oxfordshire, UK (6110 isolates). We identified eight novel *spa*-gene variants, plus one previously described. Three of these rearrangements would be designated “non-typeable” using current *spa*-typing methods; they occurred in 1.8% (72/3905) asymptomatically carried and 0.6% (14/2205) inpatient *S. aureus* strains. Some individuals were simultaneously colonized by both formerly non-typeable and typeable strains; previously such patients would have been identified as carrying only currently typeable strains, underestimating mixed carriage prevalence and diversity. Formerly non-typeable strains were found in more *spa*-types associated with multilocus sequence type ST398 (35%), common among livestock, compared to other groups with any non-typeable strains (1-4%), suggesting particular *spa*-types may have been under-represented in previous human studies.

**Conclusions:**

This improved method allows us to spa-type previously non-typeable strains with rearrangements in the spa-gene and to resolve cases of mixed colonization with deletions in one or more strains, thus accounting for hidden diversity of *S. aureus* in both community and hospital environments.

## Background

*Staphylococcus aureus* is a commensal organism that colonizes nasal mucosa in 25-30% of the healthy human population [1-6] and is responsible for a wide range of human diseases including serious nosocomial infections. *S. aureus* encodes many virulence factors including the surface Ig-binding protein A (*spa*) whose function is to capture IgG molecules in the inverted orientation and therefore prevent phagocytosis of the bacterial cells by the host immune system [7-12].

Typing the highly variable Xr region of the *spa*-gene is one of the most common methods for genotyping *S. aureus*. Even if well-established genotyping methods like MLST are indispensable, *spa*-typing has major advantages due to its high discriminatory power, typing accuracy, speed, reproducibility and ease of interpretation. *Spa*-typing also facilitates communication and data comparison between national and international clinical laboratories [13]. However, one weakness of current *spa*-typing methods is that rearrangements in the in the IgG-binding region of the gene, where the forward *spa*-primer is located, lead to 1-2% of strains being designated “non-typeable”. Five non-*spa*-typeable *S. aureus* clinical strains with rearrangements in the IgG-binding domain of the *spa*-gene were first described by Baum et al. in 2009 [14]. Although artificially constructed *spa*-deficient *S. aureus* strains are used in laboratory experiments [15-18], only a few other studies have reported variants isolated from human and cattle with rearrangements in the *spa*-gene [19-24]. Missing particular variants that cannot be typed may affect inferences about genotype associations. Whilst the prevalence of such rearrangements can be directly estimated from the proportion of non-typeable strains, detecting rearrangements that do not affect *spa*-typing would require sequencing the whole *spa*-gene; nevertheless such rearrangements may still be informative with respect to population structure.

Further complexity is introduced by the fact that most studies type only one colony per sample, thus assuming *S. aureus* colonization is by a single strain and likely systematically underestimating the number of *spa*-types per individual. The presence of non-typeable *S. aureus* strains with rearrangements in the *spa*-gene increases the number of undetected circulating *spa*-types even further.

Here we therefore developed a new set of primers to amplify the *spa*-gene from all formerly non-typeable *S. aureus* samples regardless of the specific *spa*-gene rearrangement. We used our modified *spa*-typing protocol to investigate the nature and proportion of strains with rearrangements in the *S. aureus spa*-gene in two large studies of community nasal carriers and inpatients, and the potential impact of *S. aureus* protein A mutants on epidemiological studies.

## Methods

### Collection of isolates

#### Community samples

Nasal swab samples were collected from consenting individuals recruited from five General Practices (GP) in Oxfordshire that were members of the Thames Valley Primary Care Research Partnership, National Institute of Health Research (approved by Oxfordshire Research Ethics Committee B, reference 08/H0605/102, [25]). On recruitment, a nasal swab was taken from each individual by a research nurse. Participants were trained in self-swabbing and all participants who were culture-positive for *S. aureus* (n = 360) on recruitment and one quarter (n = 211) of initially culture-negative participants were sent a self-swabbing kit after one month and then every two months. Swabs in charcoal medium were returned by mail and stored at 4°C before processing. During the 36 months of the study, *S. aureus* was isolated from at least one swab of 442 individuals yielding 3905 samples which were *spa*-typed and analyzed here.

### Inpatient samples

*S. aureus* isolates were obtained from samples collected from the Intensive care Unit (ITU), Gerontology and Trauma wards of the John Radcliffe hospital in Oxford as a part of routine screening for inpatients for infection control surveillance. For all three wards, nasal swabs were collected from individuals at ward admission and discharge as well as once a week during their stay within the ward [26]. All swabs were taken by nurses, as described above, and were processed by the routine laboratory at the John Radcliffe hospital, Oxford. In total, *S. aureus* was isolated from 2205 samples from 1273 inpatients (ITU: 1338 samples, 784 individuals; Gerontology: 134 samples, 72 individuals; Trauma: 733 samples, 417 individuals) which were *spa*-typed and analysed here.

### Isolation of *S. aureus* and DNA extraction

Each nasal swab was placed in 5% NaCl enrichment broth (E and O Laboratories) and incubated overnight at 37°C. A loopful of enrichment broth was sub-cultured onto Sa*Select* chromogenic agar (Bio-Rad) and incubated at 37°C overnight. Pink/orange colonies regarded as *S. aureus* were positively identified using a Prolex™ Staph Xtra Latex Kit (Pro-Lab Diagnostics) and catalase, DNAse and tube coagulase tests. Methicillin resistance was tested on columbia agar with 5.0% salt (Oxoid) with BBL™ Sensi-Disc™ 1 μg Oxacillin discs (BD).

Mixed glycerol stocks of *S. aureus* cultures were prepared by suspending several loopfuls of bacteria taken by sweeping across the Sa*Select* plate in 1.5 ml of saline (E and O Laboratories) with 200 μl of 45% glycerol for storage at −80°C. Taking a sweep across the plate rather than picking a single colony for glycerol stocks allowed us to maintain the genetic diversity of nasal strains in the sample for later analyses.

Crude *S. aureus* DNA extracts (‘boilates’) used for *spa*-typing were made from mixed glycerol stocks revived on Sa*Select* plates. Using a 1 mm loop, a small amount of bacteria was emulsified into 60 μl of Tris-EDTA (TE) buffer (Sigma-Aldrich), then heated in a thermocycler at 99.9°C for 10 minutes and centrifuged at 13,200 × *g* for 2 minutes. 40 μl of supernatant was removed without disturbing the pellet and stored at −20°C for use as a PCR template.

### *Spa*-typing

A staged *spa*-typing protocol was developed to enable identification of multiple-strain colonization on a large-scale [27]. The polymorphic X region of the protein A gene (*spa*) was amplified with primers 1095 F: 5′-AGACGATCCTTCGGTGAGC-3′ and 1517R: 5′-GCTTTTGCAATGTCATTTACTG-3′ [28,29]. PCR reactions consisted of 0.25 mM dNTPs (Qiagen), 0.5 U of GoTaq Flexi DNA Polymerase (Promega), Colorless GoTaq Flexi Buffer, 2.5 mM of magnesium chloride and 0.25 μM of primers in a volume of 10 μl. PCR conditions were 94°C for 2 min; 35 cycles each of 94°C for 30 s, 50°C for 30 s, and 72°C for 60 s; and a final extension at 72°C for 5 min. PCR products were purified using Agencourt AMPure XP beads (Beckman Coulter).

Samples were sequenced with the same primers as used in PCR. Sequencing reactions used BigDye v3.1 sequencing mix (Applied Biosystems) and were cycled using 30 cycles of 96°C for 10 s, 50°C for 5 s, and 60°C for 2 min. Products were purified with Agencourt CleanSEQ beads (Beckman Coulter) and separated on an ABI 3730 DNA Analyzer (Applied Biosystems).

Chromatograms were analyzed using Ridom StaphType v2.0.3 software (Ridom GmbH). The relationships between *spa*-types were investigated using the BURP clustering algorithm [30] incorporated into Ridom StaphType.

### Identification of rearrangements in *spa*-gene

**Figure 1:**
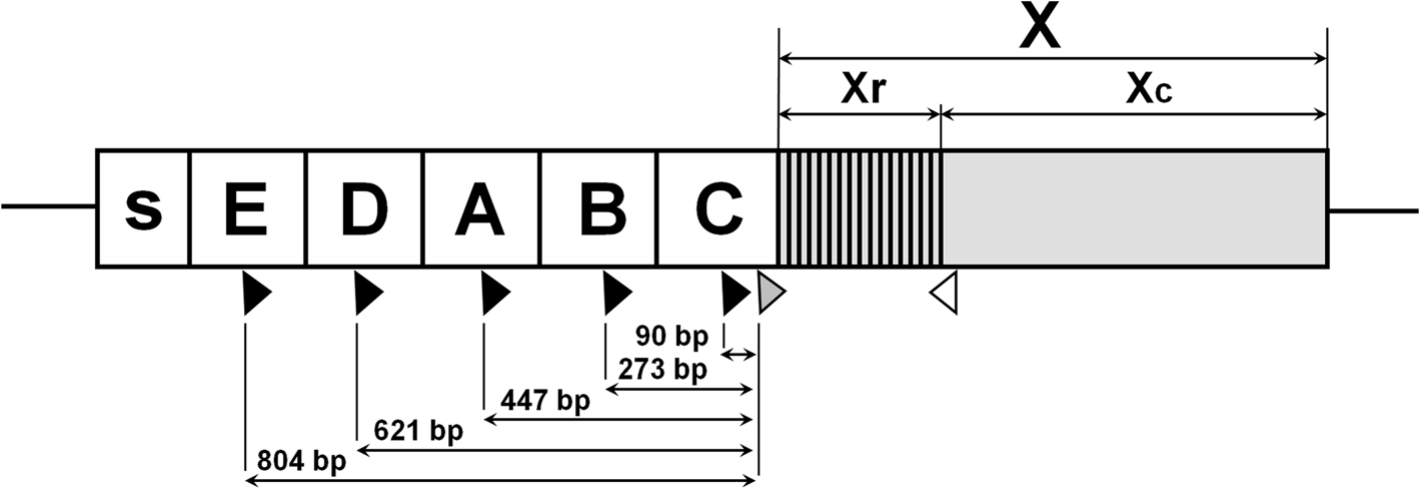
**Scheme of the*spa*-gene with annealing sites for the novel spaT3-F primer and standard primers.** Notes: black arrows indicate five annealing sites for spaT3-F primer; grey arrow indicates annealing site for 1095 F standard primer; white arrow indicates annealing site for 1517R standard primer; figures represent distance between the beginning of spaT3-F primer and the beginning of Xr region. *Spa*-gene: s – signal sequence, E, D, A, B, C – IgG-binding domains, X – region which lacks IgG-binding activity and consists of repetitive region (Xr) and C-terminal region (Xc).

A small proportion of isolates did not yield clean sequence traces with the original primers indicating the presence of rearrangements in the *spa*-gene. To identify possible rearrangements, primers spa-3 F: 5′-ATAGCGTGATTTTGCGGTT-3′ and spa-3R: 5′-CTAAATATAAATAATGTTGTCACTTGGA-3′ [14] were used to amplify the whole *spa*-gene. As some isolates failed to amplify even with this extended set of primers, an alternate forward primer, spaT3-F: 5′-CAACGCAATGGTTTCATCCA-3′ binding upstream from 1095 F was used together with standard reverse primer 1517R. Primer spaT3-F binds to a part of sequence encoding an IgG-binding domain of the *spa*-gene that is repeated five times in the gene (Figure 1). Due to presence of multiple binding sites for the spaT3-F primer within *spa*-gene, only the reverse primer (1517R) was used for sequencing.

To identify rearrangements in the *spa*-gene which do not affect standard *spa*-typing, a subset of 32 community samples and 67 bacteraemia samples were sequenced with both spa-3 F/spa-3R and spaT3-F/1517R sets of primers. 67 bacteraemia samples were randomly selected from previously existed collection of hospital invasive isolates.

Sequences were analysed using ProSeq v3.2 (http://dps.plants.ox.ac.uk/sequencing/proseq.htm).

Example sequences for each type of the *spa*-gene variant have been deposited in the GenBank under the accession numbers JX912490 to JX912498.

### Statistical analyses

Fisher’s exact test, Chi square test and 5x2 exact test were used to compare categorical variables between groups. *P* values <0.05 were considered statistically significant.

## Results and discussion

### Identification of rearrangements in the *spa*-gene

Within two large longitudinal studies of *S. aureus* carriage in the community (3905 isolates) [25] and hospital (2205 isolates) [26] several non-typeable *S. aureus* strains were identified using standard *spa*-primers (1095 F/1517R) [14]. Isolates from both studies were *spa*-typed using a staged protocol, developed to resolve single- and multiple-strain colonization [27]. According to the protocol, *spa*-sequences were classified as follows: (i) clean sequence traces were interpreted as single strain colonisation, (ii) mixed sequence traces, characterised by distinct double peaks, were interpreted as putative multiple strain colonization, and (iii) unreadable sequence traces represented failed samples, which were retyped. Samples with mixed sequence traces were further resolved by isolating 12 individual colonies; if typing of individual colonies failed, strains were considered non-typeable with standard primers.

Sequence traces of non-typeable samples showed either complete lack of amplification, or mixed sequence traces from both DNA boilates of mixed glycerol stock and of 12 individual colonies. As previously shown [14], non-typeability of *S. aureus* strains can be attributed to deletions in the *spa*-gene, explaining the lack of amplification in some of our samples. However the persistence of mixed sequence traces that could not be resolved by typing individual colonies indicated the presence of other types of *spa*-gene rearrangements.

**Figure 2:**
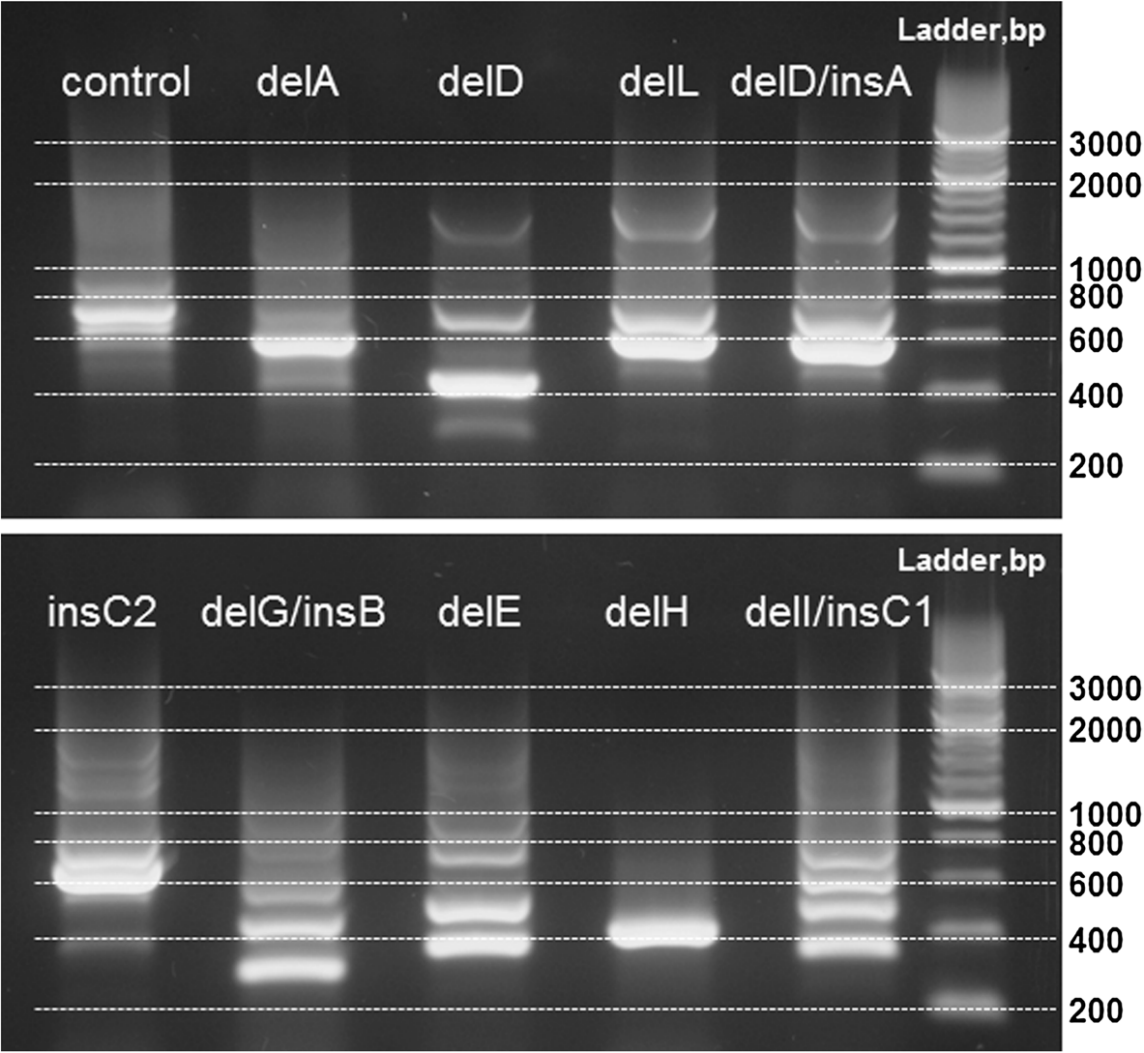
**Amplification of*spa*-locus with novel primers spaT3-F/1517R from the samples with rearrangements in the*spa*-gene.** A control sample does not have any rearrangements in the *spa*-gene and shows five PCR bands according to five annealing sites of the spaT3-F primer, including a faint band in the region of 500 bp. DelH shows one thick band most likely consisted of two merged bands from the two spaT3-F annealing sites that had been brought close together by the deletion. InsC2 has a bright band (600 bp) most likely consisted of two PCR products due to insertion of additional spaT3-F annealing site. The rest of the samples display the number of bands according to the types of rearrangements (Figure 3). Amplification of these samples with the standard *spa*-typing primers 1095 F/1517R will give no bands for the samples with delE and delG, which affect the position of 1095 F standard primer. For the rest of the sample PCR will generate a single band (double band for the insC2) located at a variable position on the ladder depending on the number of repeats within Xr region of each sample.

To identify the nature of rearrangements in all our non-typeable strains we designed a new forward spaT3-F primer and combined it with reverse primer 1517R, used for routine *spa*-typing [29]. Primer spaT3-F has a binding site in each of the five IgG-binding domains of the *spa*-gene upstream of the repetitive Xr region (Figure 1) and resulted in up to five staged PCR products per sample, depending on the type of rearrangements in the IgG-binding region (Figure 2). Due to its multisite binding within the *spa*-gene, the spaT3-F primer could be used to type samples with deletions of up to four IgG-domains of the *spa*-gene and to detect and type samples with mixtures of *S. aureus* strains with and without deletions.

With the novel spaT3-F/1517R primer set we were able to type 100% of samples that could not be *spa*-typed using the standard current set of primers (denoted “formerly non-typeable”).

**Figure 3:**
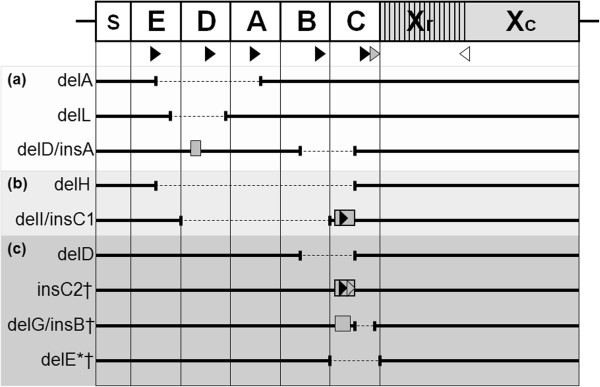
**Scheme of the rearrangements identified in the IgG-binding domains of*spa*-gene in samples from Oxfordshire.** Notes: The insertions are indicated by grey rectangles. The deletions indicated by dotted thin lines. Black arrows indicate annealing sites for spaT3-F novel primer; grey arrows indicates annealing site for 1095 F standard primer; white arrow indicates annealing site for 1517R standard primer. Grey rectangles with arrows indicate insertions with additional binding sites for primers. Panel **(a)** indicates deletions found only in community samples, panel **(b)** indicates deletions found only in inpatient samples and panel **(c)** indicates deletions found both in community and inpatient samples. *Spa* gene: s – signal sequence, E, D, A, B, C sequences encoding IgG-binding domains, X - region which lacks IgG-binding activity and consists of repetitive region (Xr) and C-terminal region (Xc). Asterisk indicates deletion previously described by Baum et al., 2009. Dagger indicates deletions/insertions leading to strains being designated non-typeable using the standard primers.

In total, we found eight completely novel deletions/insertions in the IgG-binding region of the *spa*-gene plus one deletion that has been reported before [14], in 6110 community and inpatient *S. aureus* strains from Oxfordshire (Figure 3). We never observed the deletion of the whole or a part of the repetitive Xr region in *S. aureus*, in contrast to Baum at al who described partial or total deletions of the Xr region in three bacteraemia isolates [14]: our large study suggests this happens extremely rarely in carriage. One explanation for the difference may be that Baum et al. considered disease-causing isolates while most of our community and hospital isolates were carriage.

As expected, non-typeable samples that did not amplify with the standard set of primers had one of two types of deletions affecting the binding site for the original forward primer. Deletion E (174 bp) was previously described by Baum at al. in a clinical *S. aureus* strain [14]. Deletion G (63 bp) is a novel deletion always paired with insertion B (63 bp) (Figure 3). Non-typeable samples with persistent mixed sequence traces revealed the presence of the insertion C2 (174 bp) (Figure 3). This insertion contains additional binding sites for the spaT3-F and original *spa*-forward primer, producing two PCR products and distinct double peaks in sequence traces when sequenced with the original *spa*-forward primer. Sequencing from the reverse primer (1517R) produced clean sequence traces without double peaks.

Surprisingly, in some samples that did not amplify with the standard primer set we found rearrangements represented by deletion A (357 bp) and deletion D/insertion A (174 bp/10 bp) that do not affect the position of the standard forward primer. To investigate the presence of deletions that do not affect *spa*-typing and therefore can remain unnoticed, we sequenced the whole *spa*-gene from 32 community carriage and 67 bacteraemia isolates chosen at random from the previously *spa*-typed collection. We found four novel deletions, deletion D (174 bp) in both bacteraemia and community strains, deletion L (183 bp) only in community strains, deletion H (705 bp) and deletion I/insertion C1 (531 bp/ 174 bp) only in bacteraemia isolates (Figure 3). The largest deletions of three to four IgG-binding domains were found only in *S. aureus* bacteraemia strains.

Therefore, the presence of different types of deletions and insertions in the *spa*-gene, identified by spaT3-F/1517R primers, demonstrates that *S. aureus* colonization/infection is highly complex. People may have a single strain without rearrangements, with deletions that do not affect *spa*-typing, or with rearrangements that do affect *spa*-typing. Alternatively, they may carry multiple strains without deletions in any strain, with ‘hidden’ deletions that do not affect *spa*-typing in one or more strains, or with rearrangements that do affect *spa*-typing in one or more strains.

### Prevalence of *spa*-gene rearrangements in community and hospital strains

*Spa*-typing of 3905 community *S. aureus* isolates and 2205 hospital isolates using the staged *spa*-typing protocol showed that 1.8% (n = 72) of samples from 1.8% community carriers and 0.6% (n = 14) of samples from 0.7% inpatients were formerly non-typeable (Table 1). Significantly more strains from individuals in the community were formerly non-typeable compared with hospital inpatients (p < 0.0001), and there was also a trend towards more individuals carrying formerly non-typeable strains in the community than hospital (p = 0.053). One explanation may be because more longitudinal samples were collected per individual for the community compared with the inpatient study (maximum 20 versus 4 samples respectively), providing more opportunity to encounter non-typeable strains in individuals in the community study.

**Table 1:** ***S. aureus *** **isolates with and without different types of rearrangement in the *spa *-gene in community and inpatient samples: formerly non-typeable isolates**

**Group**	**Community**^**1**^				**Hospital**^**2**^			
	**Isolates**		**Individuals**		**Isolates**		**Individuals**	
	**no.**	**%**	**no.**	**%**	**no.**	**%**	**no.**	**%**
Total	3,905	100%	442	100%	2,205	100%	1,273	100%
Pure without deletions/insertions or with hidden deletions^3^	3647	93.4%	334	75.6%	2055	93.2%	1150	90.3%
Mixed with or without deletions and/or rearrangements^4^	258	6.6%	108	24.4%	150	6.8%	123	9.7%
Formerly non-typeable: i.e. pure with rearrangements affecting standard *spa*-typing	72	1.8% (from total)	8	1.8% (from total)	14	0.6% (from total)	9	0.7% (from total)
		27.9% (from 12 picks)		7.4% (from12 picks)		9.3% (from 12 picks)		7.3% (from 12 picks)

^1^ – nasal swabs collected from individuals recruited in 5 GP practices in Oxfordshire.

^2^ – nasal swabs from individuals admitted to the adult ITU, Gerontology and Trauma wards of the Oxford University Hospitals NHS Trust.

^3^ – indicates where all samples from an individual were pure using our *spa*-typing protocol (i.e. were without deletions/insertions or only with hidden deletions) versus any sample did not fall into this category.

^4^ –subjected to 12 single colony picks, i.e. 12 sub-colonies analysed from each sample.

The proportion of *S. aureus* strains with ‘hidden’ deletions in the IgG-binging region of the *spa*-gene that do not affect *spa*-typing was estimated using spaT3-F/1517R primers on a random subset of previously typed samples. These hidden *spa*-gene deletions were found in 11% (6-19%) of *S. aureus* strains from 11% (6-19%) of individuals (Table 2).

**Table 2:** ***S. aureus *** **isolates with and without different types of rearrangement in the *spa *-gene in community and inpatient samples: isolates with hidden deletions**

**Group**	**Isolates**		**Individuals**	
	**no.**	**%**	**no.**	**%**
Total strains without deletions/insertions or with only hidden deletions investigated	99	100%	97	100%
Hidden deletions found	11	11%	11	11%

Note: Hidden deletions were found in 16% (5/32) of *S. aureus* strains from 16% (5/31) individuals in the community and in 9% (6/67) strains from 9% (6/66) hospital in patients with bacteraemia (p = 0.33); pooled data are therefore presented.

Thus up to 13% of *S. aureus* carriers could, at some point, be colonized with a strain that has deletions/insertions in the IgG-binding region of the *spa*-gene, 2% carrying completely ‘non-typeable’ strains.

### *Spa*-gene rearrangements lead mixed *S. aureus* colonization in humans to be underestimated

The staged *spa*-typing protocol allowed us to detect the simultaneous presence of two or more strains in 11% of *S. aureus* carriers. However, the presence of deletions that affect *spa*-typing in one or more strains within the mixture complicates the typing process and leads to underestimation of the prevalence of multiple colonization and number of strains involved.

The problem is illustrated using nine samples obtained over 14 months from one community nasal carrier AE (Table 3). *Spa*-typing with the standard primer sets (1095 F/1517R) demonstrated that AE only ever carried one strain, which was type t230 at most time points, the *spa*-type t008 on one occasion and one non-typeable strain. Re-typing the same DNA extractions with our alternative novel primers (spaT3-F/1517R) showed that all samples had mixed sequence traces, apart from the formerly non-typeable strain that had deletion E associated with *spa*-type t012. Therefore, 12 single colonies were isolated for each sample and re-typed with alternative primers. This identified five *spa*-types carried by AE at various time points, and mixed strain colonization by two-three *spa*-types on four occasions, including two strains with deletion E. We were unable to resolve all samples by typing 12 individual colonies, even though they showed presence of mixed sequence traces (time points 4, 10, 12 and 14), which could be explained by a low frequency of one of the colonizing strains.

**Table 3:** ***Spa*-typing of *S. aureus *** **strains from a single individual AE with two sets of primers: standard primers 1095 F/1517R and novel primers spaT3-F/1517R**

**Time points, months**	**DNA prep (mixed boilate)**		**12 single colony picks**^**2**^
	**1095 F/1517R**	**spaT3-F/1517R**	**spaT3-F/1517R**
AE-0	t230	MST^1^	t230/**t012**
AE-1	non-typable	**t012**	**t012**
AE-2	t230	MST	t230/**t012**
AE-4	t230	MST	t230
AE-6	t230	MST	t230/t528
AE-8	t008	MST	t008/**t012/t571**
AE-10	t230	MST	t230
AE-12	t230	MST	t230
AE-14	t230	MST	t230

^1^mixed sequence traces; ^2^*spa*-types in bold have delE and could not be typed with standard primers; spa-repeats:

t230: 08-16-02-16-34.

t571: 08-16-02-25-02-25-34-25.

t008: 11-19-12-21-17-34-24-34-22-25.

t012: 15-12-16-02-16-02-25-17-24-24.

t528: 04.

The limitations of the conventional *spa*-typing protocol make it impossible to identify and type *S. aureus* strains with rearrangements in the *spa*-gene in individuals with multiple strain colonization. The staged *spa*-typing protocol allows us to resolve cases of mixed strain colonization with deletions in one or more strains. Even 12 single colony picks could not always identify the presence of low-frequency strains with deletions, illustrating the even greater challenge of estimating the proportion of non-typeable strains within mixed colonization. Thus diversity in colonizing and infecting strains is inevitably underestimated.

### Inpatients’ strains can acquire deletions in the *spa*-gene

We also found that *S. aureus* strains can acquire deletions in the *spa*-gene during inpatients’ hospital admission. Such acquisition of deletions was never observed for longitudinal carriage strains from individuals in the community, with those deletions observed being present from the first time the strain carrying the deletion was identified (Additional file 1: Table S1).

Among six hospital patients with deletions that affect *spa*-typing, three individuals (BA, BB and BF) already carried the strain with the deletion when they were admitted. The three other patients (BC, BD and BE) acquired a deletion during their hospital stay on the background of a *spa*-type carried at admission (Table 4). There were eight, four and six days between the last swab without and the first swab with the acquired deletion for BC, BD and BE respectively. All three patients acquiring deletions during hospital admission either had long-term illnesses and/or had taken several antibiotics (BC: teicoplanin; BD: doxycycline; BD: flucloxacillin, penicillin, ciprofloxacin, vancomycin, erythromycin, gentamicin, tetracycline).

**Table 4:** **Individuals who acquired a deletion in the *S. aureus spa *-gene during their hospital admission**

**Individual ID**	**Date swab taken**	**Results**	**Spa type**	**Rearrangements**
BC	30/01/2011	MSSA	t298	
BC	08/02/2011	MSSA	**t298**	delG-insB
BD	14/04/2011	MSSA	t571	
BD	19/04/2011	MSSA	**t571**	delG-insB
BD	26/04/2011	MSSA	**t571**	delG-insB
BE-a^1^	20/06/2011	MSSA	t179	
BE-g^2^	20/06/2011	MSSA	t179	
BE-n^3^	20/06/2011	MSSA	t179/t078	
BE-th^4^	20/06/2011	MSSA	t179/t078	
BE	05/07/2011	MSSA	t179/t078	
BE	12/07/2011	MSSA	t179/**t078**	delE
BE	20/07/2011	MSSA	t179/**t078**	delE

^1–4^body sites swabs: a – axilla, g – groin, n – nose, th – throat; all other swabs are nasal swabs; *spa*-types in bold acquired deletion that affects binding site for standard forward *spa*-typing primer.

The repetitive nature of the *spa*-gene makes it unstable and highly prone to internal rearrangements, which in bacteria occur via either RecA-dependent or RecA-independent recombination [31-33]. These rearrangements might have positive or negative effects as protein A is an important virulence factor that plays a central role in *S. aureus* defence against the host immune response. There is new evidence that the antibiotic ciprofloxacin increases the intrachromosomal DNA recombination rate in *Escherichia coli*[34]. Other antibiotics might potentially have similar effects, yet undiscovered. Taking into account that the three inpatients who acquired deletions during their stay at the hospital had been taking specific antibiotics for a long time or a wide range of antibiotics for a short period, including ciprofloxacin, it is possible that antibiotic pressure might be one factor that drives genetic rearrangements in the *S. aureus* protein A gene. However, we also cannot exclude the possibility that these deletions may have been present already at low frequency, and undetected, before increasing to become the majority variant (rather than being acquired de novo). Nevertheless this scenario also would support antibiotics playing a role in emergence of deletions to detectable levels.

In the community, most individuals colonized by *S. aureus* strains carry them without displaying any symptoms. However, when some of them became invasive, the change of habitat, for example on a background of antibiotic pressure, might promote acquisition of rearrangements in the *spa*-gene that might be advantageous in new environment even if they lead to loss or change of protein function. As shown previously, progress of infectious disease is typically associated with acquisition of mutations or rearrangements in the pathogen, many of which lead to loss of function in a number of genes [35].

### Deletions appear to be over-represented in clonal lineages related to livestock

In total, we found 20 *spa*-types from 33 individuals associated with nine types of rearrangements in the *spa*-gene (Additional file 2: Table S2). All types of deletions were associated with a mixture of related and unrelated *spa*-types, only insertion C2 was associated with a group of 3 closely related *spa*-types: t021, t012 and t10173. The 20 *spa*-types with rearrangements were clustered into five groups of closely-related variants and five non-related singletons (Table 5).

**Table 5:** ***Spa*-types and groups in which deletions/insertions in the *spa *-gene were observed**

***Spa*-types**	***Spa*-repeats**	**Individuals with deletions, no. (column %)**	**Hidden deletions not affecting *spa *-typing (no.)**	**Deletions/insertions affecting *spa *-typing (no.)**	**Individuals with deletions affecting *spa- *** **typing/total individuals with this spa-type**
Group 1		7 (21%)			7/20 (35%)*
t571	08-16-02-25-02-25-----------34-25	6 (18%)		delG-insB(5); delE(1)	6/19 (32%)
t3085	08-16-02-25-02-25-34-25-34-25	1 (3%)		delE (1)	1/1 (100%)
Group 2		9 (27%)			7/188 (4%)*
t021	15-12------16-02-16---------------02-25-17-24--------------	4 (12%)	delD(1)	insC2(3)	3/57 (5%)
t298	15-12------16-02-----------------------------17-24--------------	1 (3%)		delG-insB (1)	1/5 (20%)
t10173	15-12-02-16-02------25-17-25-02-25-17-24-24---------	1 (3%)		insC2 (1)	1/1 (100%)
t012	15-12------16-02-16---------------02-25-17-24-24---------	2 (6%)		delE (1); insC2 (1)	2/123 (2%)
t6803	15-12------16-02-16---------------02-25-17-24-24-17-24	1 (3%)	delD-insA (1)		0/2 (0%)
Group 3		3 (9%)			-
t084	07-23-12-34-34-12-12-23-02-12-23	1 (3%)	delH (1)		-
t085	07-23-12-34-34-12-----23-02-12-23	2 (6%)	delD (1); delA (1)		-
Group 4		4 (12%)			3/74 (4%)*
t280	04--------------------20-17-12-12-17-------------	1 (3%)		delG-insB (1)	1/4 (25%)
t227	04-----------------------------12-12-17-------------	1 (3%)	delD (1)		0/3 (0%)
t078	04-21^a^-12^b^-41^c^-20-17-12-12-17-------------	1 (3%)		delE (1)	1/26 (4%)
t216	04----------20-17-20-17------------31^d^-16^e^-34^f^	1 (3%)		delG-insB (1)	1/41 (2%)
Group 5		3 (9%)			1/92 (1%)*
t032	26-23-23-13-23-31-29-17-31-29-17-25-17-25-16-28	2 (6%)	delD (1)	delE (1)	1/79 (1%)
t223	26-23-----13-23-------------------05^g^-17-25-17-25-16-28	1 (3%)	delD (1)		0/13 (0%)
Singletons		7 (21%)			-
t213	07-23-12-21-24-33-22-17	3 (9%)	delD (3)		-
t6792	08-16-02-16-17-13-17-13-17-16-34	1 (3%)	delD (1)		-
t6417	14-44-13-12-17-13-12-17-17-17-23-18	1 (3%)	dell (1)		-
t530	11-19-12-21-17-34-24-34-16	1 (3%)		delE (1)	1/3 (33%)*
t7960	299-25-17-17-16-16-16-16	1 (3%)	delI-insC1 (1)		-
Total		33 (100%)			

* P < 0.0001 comparing four groups of spa-types and the singleton with rearrangements affecting spa-typing (5 × 2 Fisher’s exact test).

21^a^ repeat differing in one base from repeats 12 and 20.

12^b^ repeat differing in two bases from repeats 20.

41^c^ repeat differing in one base from repeat 17.

31^d^ repeat differing in two bases from repeat 16.

16^e^ repeat differing in one base from repeat 17.

34^f^ repeat differing in two bases from repeats 12 and 20.

05^g^ repeat differing in one base from repeat 29.

Any clusters of related *spa*-types with a higher prevalence of rearrangements affecting *spa*-typing (delE, delG-insB or insC2) would be likely to be underrepresented, or even missing, in the majority of studies based on routine *spa*-typing protocols. To test this hypothesis, we compared the proportion of individuals with and without rearrangements affecting *spa*-typing in the four groups of *spa*-types and the singleton that we found had one or more rearrangements affecting *spa*-typing (5 × 2 exact test, Table 5). In group 1 35% of strains were affected by these rearrangements, a significantly higher proportion compared with the 1-4% in other groups or the singleton t530 (p < 0.0001). Therefore, *spa*-type t571 and its closely related variants such as t3085 may well be underrepresented in most *S. aureus* studies based on *spa*-typing, as they could not be typed with the standard set of primers when common deletions are present.

Interestingly, *spa*-type t571 belongs to clonal lineage ST398 that contains MRSA and MSSA strains common among livestock. *Spa*-type t571 is closely related to type t011 and t034, all most commonly associated with pigs [36-40]. These *spa*-types have been found less commonly in dogs, cats and horses, and occasionally in cattle and poultry [41,42]. Large-scale screening of pigs [36] showed that 60% of them carried t034, 14% t1255 and 1.5% t571. Although ST398-associated *spa*-types have been rarely found among the general human population, they have been found more commonly in farmers working with pigs [36,37]. Veterinary personnel and pet owners are also more likely to carry these animal-related types [43]. Recent studies have also reported the emergence of livestock-associated MRSA clones of *S. aureus* ST398 causing bacteraemias in humans, supporting animal-independent transmission of such strains between humans [44,45].

It is unclear why ST398 *S. aureus* strains commonly found in livestock frequently develop deletions in the IgG-binding part of protein A gene after transmission to humans. One possible explanation is that this might be a part of *S. aureus* strain adaptation to a different immune background where protein A plays a major role [7,8,12]. Another explanation might be that the livestock associated strains have more rearrangements in the *spa*-gene prior the transmission to humans due to high level of antibiotic exposure in food-animal production [46-48]. Nevertheless, our findings highlight the potential for these strains to have been substantially under-represented in epidemiological studies to date, and for strains formerly not-typeable using standard methods to be a source of bias.

## Conclusions

*Spa*-typing is a common technique used to classify *S. aureus* strains in clinical practice (eg outbreak management) and research. Rearrangements in the IgG-binding region of the *spa*-gene make strains “non-typeable” with commonly used primers. Using a novel primer, we typed 100% of samples and identified eight novel *spa*-gene variants, plus one previously described; three of these rearrangements cause strains to be designated as “non-typeable” using current *spa*-typing methods.

*Spa*-typing of 6110 *S. aureus* isolates showed that 1.8% of samples from 1.8% community carriers and 0.6% of samples from 0.7% inpatients were formerly non-typeable. We also found evidence of mixed colonization with strains with and without gene rearrangements, and estimated that up to 13% of carriers are colonized with “hidden” *S. aureus* with deletions/insertions in the IgG-binding region at some point. Using standard primers therefore underestimates *spa*-type diversity. We also found evidence of inpatients acquiring spa-gene deletions de novo during a hospital admission, suggesting that antibiotic pressure might be one factor driving genetic rearrangements in the *S. aureus* protein A gene. Finally, we found that deletions formerly causing strains to be designated as “non-typeable” were over-represented in clonal lineages related to livestock, indicating that these may well be have been underrepresented in most *S. aureus* studies. This new improved spa-typing protocol therefore enables previously overlooked *S. aureus* strains to be typed and therefore contribute to our understanding of diversity, carriage and transmission of *S. aureus* strains in community and hospitals.

## Competing interests

All authors declare that they have no competing interests.

## Authors’ contributions

AAV carried out laboratory experiments, participated in the analysis of data and writing of the manuscript, RF contributed to the collection and processing of samples for the study, RRM contributed to the design of the sample collection and sample database development, KK supervised recruitment of participants, as well as collection and processing of samples for the study, HG recruited participants for the study, DHW contributed in the design of the study and laboratory experiments, RB participated in the design of the study, DWC participated in the design of the study and contributed in the drafting of the manuscript, ASW performed statistical analysis and participated in the writing of the manuscript. All authors read and approved the final manuscript.

## Supplementary Material

Additional file 1: Table S1 Swab data for individuals with rearrangements in the *spa*-gene. Click here for file

Additional file 2: Table S2 Association between rearrangements in the *spa*-gene and *spa*-types. Click here for file
