# Supplementary material for: Prevalence of Staphylococcus aureus protein A (spa) mutants in the community and hospitals in Oxfordshire
Source: BMC Microbiol. 2014 Mar 12;14:63. doi: 10.1186/1471-2180-14-63 (PMC4007515; doi:10.1186/1471-2180-14-63)
Supplement: Additional file 1: Table S1 — Swab data for individuals with rearrangements in the spa-gene. [file 1471-2180-14-63-S1.pdf]

Supplementary Table 1: Swab data for individuals with rearrangements in the *spa*-gene

Participants from community carriage study				
Individual ID	Month swab taken	Results	*Spa*-type	Rearrangements
AA-0	0	no growth		
AA-1	1	MSSA	t1166	
AA-2	2	no growth		
AA-4	4	MSSA	t1166/**t571**	delG-insB
AA-6	6	MSSA	t1166	
AA-8	8	no growth		
AA-10	10	no growth		
AA-12	12	no growth		
AA-14	14	MSSA	t1166	
AA-16	16	MSSA	t1166	
AA-18	18	no growth		
AA-20	20	no growth		
AA-22	22	MSSA	t1166	
AA-24	24	no growth		
AA-26	26	no growth		
AA-28	28	no growth		
AA-30	30	no growth		
AA-32	32	no growth		
AB-0	0	MSSA	t084/**t280**	delG-insB
AB-1	1	MSSA	**t280**	delG-insB
AB-2	2	MSSA	**t280**	delG-insB
AB-4	4	MSSA	**t280**	delG-insB
AB-6	6	MSSA	**t280**	delG-insB
AB-8	8	MSSA	**t280**	delG-insB
AB-10	10	MSSA	**t280**	delG-insB
AB-12	12	MSSA	**t280**	delG-insB
AB-14	14	MSSA	**t280**	delG-insB
AB-16	16	MSSA	**t280**	delG-insB
AB-18	18	MSSA	**t280**	delG-insB
AB-20	20	MSSA	**t280**	delG-insB
AB-22	22	MSSA	**t280**	delG-insB
AB-24	24	MSSA	**t280**	delG-insB
AB-26	26	MSSA	**t280**	delG-insB
AB-28	28	MSSA	**t280**	delG-insB
AB-30	30	MSSA	**t280**	delG-insB
AB-32	32	MSSA	**t280**	delG-insB
AC-0	0	MSSA	**t530**/t630	delE
AC-1	1	MSSA	**t530**/t084	delE
AC-2	2	MSSA	**t530**/t084	delE
AC-4	4	not returned		
AC-6	6	MSSA	**t530**	delE
AC-8	8	MSSA	**t530**	delE
AC-10	10	MSSA	**t530**	delE
AC-12	12	MSSA	**t530**	delE
AC-14	14	MSSA	**t530**	delE
AC-16	16	MSSA	**t530**	delE
AC-18	18	MSSA	**t530**	delE
AC-20	20	not returned		
AC-22	22	not returned		
AC-24	24	MSSA	**t530**	delE
AC-26	26	MSSA	**t530**	delE

AC-28	28	MSSA	**t530**	delE
AC-30	30	MSSA	**t530**	delE
AC-32	32	MSSA	**t530**	delE
AD-0	0	no growth		
AD-1	1	no growth		
AD-2	2	no growth		
AD-4	4	no growth		
AD-6	6	no growth		
AD-8	8	MSSA	**t3085**	delE
AD-10	10	no growth		
AD-12	12	no growth		
AD-14	14	MSSA	**t3085**	delE
AD-16	16	MSSA	**t3085**	delE
AD-18	18	MSSA	**t3085**	delE
AD-20	20	MSSA	t400	
AD-22	22	MSSA	t5973	
AD-24	24	MSSA	**t3085**	delE
AE-0	0	MSSA	t230/**t012**	delE
AE-1	1	MSSA	**t012**	delE
AE-2	2	MSSA	t230/**t012**	delE
AE-4	4	MSSA	t230	
AE-6	6	MSSA	t230/t528	
AE-8	8	MSSA	t008/**t012**/**t571**	delE/delG-insB
AE-10	10	MSSA	t230	
AE-12	12	MSSA	t230	
AE-14	14	MSSA	t230	
AE-16	16	MSSA	t230	
AE-18	18	MSSA	t230	
AE-20	20	MSSA	t230	
AE-22	22	MSSA	t230	
AE-24	24	MSSA	t230	
AF-AD		MRSA	**t032**	delE
AF-DI		MRSA	**t032**	delE
AF-0	0	MRSA	**t032**/t209	delE
AF-1	1	not returned		
AF-2	2	MSSA	t021	
AF-4	4	MSSA	t021	
AF-6	6	not returned		
AG-0	0	MSSA	t364	
AG-1	1	MSSA	**t021**	insC2
AG-2	2	MSSA	**t021**	insC2
AG-4	4	MSSA	**t021**	insC2
AG-6	6	MSSA	**t021**	insC2
AG-8	8	MSSA	**t021**	insC2
AG-10	10	MSSA	**t021**	insC2
AG-12	12	MSSA	**t021**	insC2
AG-14	14	MSSA	**t021**	insC2
AG-16	16	MSSA	**t021**	insC2
AG-18	18	MSSA	**t021**	insC2
AG-20	20	MSSA	**t021**	insC2
AG-22	22	MSSA	**t021**	insC2
AG-24	24	MSSA	**t021**	insC2
AH-0	0	MSSA	**t021**	insC2
AH-1	1	MSSA	**t021**	insC2
AH-2	2	MSSA	**t021**	insC2
AH-4	4	MSSA	**t021**	insC2

AH-6	6	MSSA	**t021**	insC2
AH-8	8	MSSA	**t021**	insC2
AH-10	10	MSSA	**t021**	insC2
AH-12	12	MSSA	**t021**	insC2
AH-14	14	not returned		
AH-16	16	MSSA	**t021**	insC2
AH-18	18	MSSA	**t021**	insC2
AH-20	20	MSSA	**t021**	insC2
AH-22	22	MSSA	**t021**	insC2
AH-24	24	MSSA	**t021**	insC2
AI-0	0	MSSA	t227	delD
AI-1	1	MSSA	t227	delD
AI-2	2	MSSA	t227	delD
AI-4	4	MSSA	t227	delD
AI-6	6	MSSA	t227	delD
AI-8	8	MSSA	t227	delD
AI-10	10	MSSA	t227	delD
AI-12	12	MSSA	t227	delD
AI-14	14	MSSA	t227	delD
AI-16	16	MSSA	t227	delD
AI-18	18	MSSA	t227	delD
AI-20	20	MSSA	t227	delD
AI-22	22	MSSA	t227	delD
AI-24	24	not returned		
AJ-0	0	MSSA	t085	delD
AJ-1	1	MSSA	t085	delD
AJ-2	2	MSSA	t085	delD
AJ-4	4	MSSA	t085	delD
AJ-6	6	MSSA	t085	delD
AJ-8	8	MSSA	t085	delD
AJ-10	10	not returned		
AJ-12	12	MSSA	t085	delD
AJ-14	14	MSSA	t085	delD
AJ-16	16	not returned		
AJ-18	18	not returned		
AJ-20	20	not returned		
AK-0	0	MSSA	t213	delD
AK-1	1	MSSA	t213	delD
AK-2	2	MSSA	t213	delD
AK-4	4	MSSA	t213	delD
AK-6	6	MSSA	t213	delD
AK-8	8	MSSA	t213	delD
AK-10	10	MSSA	t213	delD
AK-12	12	MSSA	t213	delD
AK-14	14	MSSA	t213	delD
AK-16	16	MSSA	t213	delD
AK-18	18	MSSA	t213	delD
AK-20	20	not returned		
AL-0	0	MSSA	t6792	delD
AL-1	1	MSSA	t6792	delD
AL-2	2	MSSA	t6792	delD
AL-4	4	MSSA	t6792	delD
AL-6	6	MSSA	t6792	delD
AL-8	8	MSSA	t6792	delD
AL-10	10	MSSA	t6792	delD
AL-12	12	MSSA	t6792	delD

AL-14	14	MSSA	t6792	delD
AL-16	16	MSSA	t6792	delD
AL-18	18	MSSA	t6792	delD
AL-20	20	MSSA	t6792	delD
AL-22	22	MSSA	t6792	delD
AL-24	24	MSSA	t6792	delD
AL-26	26	MSSA	t6792	delD
AL-28	28	MSSA	t6792	delD
AL-30	30	MSSA	t6792/t10237	delD/no deletion
AL-32	32	MSSA	t6792	delD
AL-34	34	MSSA	t6792	delD
AL-36	36	MSSA	t6792	delD
AM-0	0	MSSA	t659	
AM-1	1	MSSA	t659/t085	
AM-2	2	MSSA	t659	
AM-4	4	MSSA	t659/t6417	no deletion/delL
AM-6	6	MSSA	t6417	delL
AM-8	8	MSSA	t6417	delL
AM-10	10	MSSA	t6417	delL
AM-12	12	MSSA	t6417	delL
AM-14	14	MSSA	t6417	delL
AM-16	16	MSSA	t6417	delL
AM-18	18	MSSA	t6417	delL
AM-20	20	MSSA	t6417	delL
AM-22	22	MSSA	t6417	delL
AM-24	24	MSSA	t6417	delL
AM-26	26	MSSA	t6417	delL
AM-28	28	no growth		
AM-30	30	no growth		
AM-32	32	no growth		
AM-34	34	no growth		
AM-36	36	no growth		
AN-0	0	no growth		
AN-1	1	not returned		
AN-2	2	MSSA	t085	delA
AN-4	4	not returned		
AN-6	6	no growth		
AN-8	8	no growth		
AN-10	10	not returned		
AN-12	12	no growth		
AN-14	14	not returned		
AN-16	16	no growth		
AN-18	18	no growth		
AN-20	20	no growth		
AN-22	22	no growth		
AN-24	24	not returned		
AO-0	0	MSSA	t6803	delD-insA
AO-1	1	MSSA	t6803	delD-insA
AO-2	2	MSSA	t6803	delD-insA
AO-4	4	MSSA	t6803	delD-insA
AO-6	6	MSSA	t6803	delD-insA
AO-8	8	MSSA	t6803	delD-insA
AO-10	10	MSSA	t6803	delD-insA
AO-12	12	MSSA	t6803	delD-insA
AO-14	14	MSSA	t6803	delD-insA
AO-16	16	MSSA	t6803	delD-insA

AO-18	18	MSSA	t6803	delD-insA
AO-20	20	MSSA	t6803	delD-insA
AO-22	22	MSSA	t6803	delD-insA
AO-24	24	MSSA	t6803	delD-insA
AO-26	26	MSSA	t6803	delD-insA
AO-28	28	MSSA	t6803	delD-insA
AO-30	30	MSSA	t6803	delD-insA
AO-32	32	MSSA	t6803	delD-insA
AO-34	34	MSSA	t6803	delD-insA
AO-36	36	MSSA	t6803	delD-insA

Inpatients from the Oxford University Hospitals NHS Trust

Swab ID	Date taken	Results	Spa type	Rearrangements
BA	08/05/2010	MSSA	**t571**	delG-insB
BB	24/10/2010	MSSA	**t216**	delG-insB
BC	30/01/2011	MSSA	t298	
BC	08/02/2011	MSSA	**t298**	delG-insB
BD	14/04/2011	MSSA	t571	
BD	19/04/2011	MSSA	**t571**	delG-insB
BD	26/04/2011	MSSA	**t571**	delG-insB
BE-a[1]	20/06/2011	MSSA	t179	
BE-g[2]	20/06/2011	MSSA	t179	
BE-n[3]	20/06/2011	MSSA	t179/t078	
BE-th[4]	20/06/2011	MSSA	t179/t078	
BE	05/07/2011	MSSA	t179/t078	
BE	12/07/2011	MSSA	t179/**t078**	delE
BE	20/07/2011	MSSA	t179/**t078**	delE
BF	24/09/2011	MSSA	**t571**	delE
BF	24/09/2011	MSSA	**t571**	delE
BG	29/12/2010	MSSA	t10173	insC2
BH	25/02/2011	MSSA	t021	insC2
BH	01/03/2011	MSSA	t021	insC2
BI	14/05/2011	MSSA	t012	insC2
BI	17/05/2011	MSSA	t012	insC2

Oxford inpatients with bacteraemia

Swab ID	Date taken	Results	Spa type	Rearrangements
CA	24/02/2009	MRSA	t032	delD
CB	11/02/2009	MSSA	t021	delD
CC	15/02/2010	MSSA	t084	delH
CD	24/02/2009	MSSA	t213	delD
CE	10/01/2009	MSSA	t213	delD
CF	10/04/2009	MSSA	t223	delD
CG	03/11/2009	MSSA	t7960	delI-insC1

[1-4] body sites swabs: a – axilla, g – groin, n – nose, th – throat; all other swabs are nasal swabs; *spa*-types in bold have deletions that affect binding site for forward *spa*-typing primer
