# Supplementary material for: Prevalence of Staphylococcus aureus protein A (spa) mutants in the community and hospitals in Oxfordshire
Source: BMC Microbiol. 2014 Mar 12;14:63. doi: 10.1186/1471-2180-14-63 (PMC4007515; doi:10.1186/1471-2180-14-63)
Supplement: Additional file 2: Table S2 — Association between rearrangements in the spa-gene and spa-types. [file 1471-2180-14-63-S2.pdf]

Supplementary Table 2: Association between rearrangements in the *spa*-gene and *spa*-types

| Deletions/ Insertions | *Spa*-types | *Spa*-repeats | No. of *spa*-types | No. of individuals | No. (%) of individuals |
|---|---|---|---|---|---|
| delA | t085 | 07-23-12-34-34-12-----23-02-12-23 | 1 | 1 | 1 (3%) |
| delD | t032 | 26-23-23-13-23------31-29-17-31-29-17-25-17-25-16-28 | 7 | 1 | 9 (27%) |
| | t223 | 26-23------13-23-05-----------------------17-25-17-25-16-28 | | 1 | |
| | t021 | 15-12-16-02-16-02-25-17-24 | | 1 | |
| | t227 | 04-12-12-17 | | 1 | |
| | t085 | 07-23-12-34-34-12-23-02-12-23 | | 1 | |
| | t213 | 07-23-12-21-24-33-22-17 | | 3 | |
| | t6792 | 08-16-02-16-17-13-17-13-17-16-34 | | 1 | |
| delD-insA | t6803 | 15-12-16-02-16-02-25-17-24-24-17-24 | 1 | 1 | 1 (3%) |
| delE | t571 | 08-16-02-25-02-25----------34-25 | 6 | 1 | 6 (18%) |
| | t3085 | 08-16-02-25-02-25-34-25-34-25 | | 1 | |
| | t530 | 11-19-12-21-17-34-24-34-16 | | 1 | |
| | t032 | 26-23-23-13-23-31-29-17-31-29-17-25-17-25-16-28 | | 1 | |
| | t012 | 15-12-16-02-16-02-25-17-24-24 | | 1 | |
| | t078 | 04-21-12-41-20-17-12-12-17 | | 1 | |
| delG-insB | t280 | 04-20-17-12-12-17---------------------- | 4 | 1 | 8 (24%) |
| | t216 | 04-20-17--------------20-17-31-16-34 | | 1 | |
| | t571 | 08-16-02-25-02-25-34-25 | | 5 | |
| | t298 | 15-12-16-02-17-24 | | 1 | |
| delH | t084 | 07-23-12-34-34-12-12-23-02-12-23 | 1 | 1 | 1 (3%) |
| delI-insC1 | t7960 | 299-25-17-17-16-16-16-16 | 1 | 1 | 1 (3%) |
| delL | t6417 | 14-44-13-12-17-13-12-17-17-17-23-18 | 1 | 1 | 1 (3%) |
| insC2 | t021 | 15-12-----16-02-16--------------02-25-17-----24 | 3 | 3 | 5 (15%) |
| | t012 | 15-12-----16-02-16--------------02-25-17-24-24 | | 1 | |
| | t10173 | 15-12-02-16-02-----25-17-25-02-25-17-24-24 | | 1 | |
| Total: | 20* | | | 33 | |

* Note: *spa*-types t085, t571, t021, t012, t032 occur with two different types of deletions
